# Mortality Statistics, Chicago

**Published:** 1875-02

**Authors:** 


***Chicago Mortality Report for December, 1874.*** Reported by Dr. Ben. C. Miller, Sanitary Superintendent.

| | |
|---|---|
| Accident, crushed | 1 |
| " by chloroform | 1 |
| " by explosion | 1 |
| " by burns | 1 |
| " by fall | 5 |
| " fracture of skull | 1 |
| " by suffocation | 1 |
| " by scalding | 4 |
| " by railroad | 2 |
| " by street cars | 1 |
| Anæmia | 1 |
| Apoplexy | 7 |
| Asphyxia | 1 |
| Asthma | 4 |
| Ascitis | 2 |
| Bowels, congestion of | 1 |
| " hemorrhage of | 1 |
| Brain, congestion of | 7 |
| " disease of | 1 |
| " inflammation of | 7 |
| Bronchitis | 12 |
| Bronchitis, capillary | 2 |
| Cancer | 2 |
| " of bowels | 1 |
| " of jaw bone | 1 |
| " of stomach | 2 |
| " of uterus | 1 |
| Cholera infantum | 1 |
| Consumption | 54 |
| Convulsions | 55 |
| " uræmic | 1 |
| Croup | 12 |
| " membranous | 5 |
| Debility, general | 4 |
| Delirium tremens | 4 |
| Diarrhœa | 3 |
| " chronic | 1 |
| Diphtheria | 7 |
| Dropsy, general | 8 |
| " pericardium | 1 |
| Dysentery | 2 |
| Embolism | 1 |
| Endo carditis | 1 |
| Enteritis | 15 |
| Entero-colitis | 1 |
| Encephaloid disease of lungs | 1 |
| Erysipelas | 4 |
| Exhaustion | 1 |
| Fever, puerperal | 7 |
| " scarlet | 7 |
| " " malignant | 3 |
| " typhoid | 21 |
| Gangrene | 1 |
| Gastro enteritis | 1 |
| Heart, aneurism of | 1 |
| Heart, disease of | 5 |
| " organic disease of | 1 |
| " hypertrophy of | 2 |
| " mitral valves, disease of | 1 |
| " malformation of | 1 |
| " valvular disease of | 5 |
| Hemiplegia | 1 |
| Hemorrhage, cerebral | 1 |
| Hernia, inguinal | 1 |
| " incarcerated | 1 |
| Hydrophobia | 1 |
| Hydrocephalus | 5 |
| Inanition | 11 |
| Inflammation following surg. op'r'n. | 1 |
| Intemperance | 2 |
| Intussusception | 2 |
| Jaundice | 1 |
| Kidneys, Bright's disease of | 5 |
| " inflammation of | 1 |
| Liver, disease of | 3 |
| " cirrhosis of | 2 |
| " tumor of | 1 |
| " stricture of | 1 |
| Lungs, abscess of | 1 |
| " congestion of | 5 |
| " paralysis of | 1 |
| Malformation | 2 |
| Meningitis | 3 |
| " cerebro-spinal | 5 |
| " tubercular | 2 |
| Œdema, pulmonum | 1 |
| Old age | 11 |
| Paralysis | 4 |
| Peritonitis | 8 |
| " puerperal | 1 |
| Pneumonia | 31 |
| " typhoid | 2 |
| Pleurisy | 1 |
| Pyæmia | 2 |
| Sarcoma of thigh | 1 |
| Scurvy | 1 |
| Septicæmia | 1 |
| Spleen, liver & lungs, congest'n of | 1 |
| Stone in bladder | 1 |
| Strangulation | 1 |
| Stomatitis | 1 |
| Syphilis | 1 |
| Tabes mesenterica | 7 |
| Uterus, hemorrhage of | 2 |
| Whooping cough | 1 |
| Suicide, by poison | 2 |
| " by cutting throat | 1 |
| " by morphine | 1 |
| Total | 451 |

Premature births, 6; Still births, 56. Total, 62.

COMPARISON.

Deaths in December, 1874, 451; in November, 1874, 440. Increase, 11. Deaths in December, 1873, 566. Decrease, 115.

AGES.

| | | | |
|---|---|---|---|
| Under one year | 129 | Thirty years to forty | 49 |
| One year to two | 26 | Forty " " fifty | 39 |
| Two years to three | 19 | Fifty " " sixty | 35 |
| Three " " four | 12 | Sixty " " seventy | 20 |
| Four " " five | 6 | Seventy " " eighty | 16 |
| Five " " ten | 14 | Eighty " " ninety | 5 |
| Ten " " twenty | 16 | Ninety " " one hundred | 2 |
| Twenty " " thirty | 63 | Total | 451 |
| Males | 238 | Colored | 11 |
| Females | 213 | White | 440 |
| Total | 451 | Total | 451 |

Married, 175; Single, 276. Total, 451.

NATIVITIES.

| | | | | | |
|---|---|---|---|---|---|
| Austria | 2 | Denmark | 4 | Norway | 5 |
| Bohemia | 1 | England | 10 | Scotland | 4 |
| Canada | 4 | Germany | 74 | Sweden | 7 |
| Native—Chicago | 57 | Holland | 4 | Switzerland | 1 |
| Foreign, " | 126 | Ireland | 55 | Wales 1 Unknown 7 | 8 |
| U. States, other parts | 88 | Italy | 1 | Total | 451 |

Deaths daily, 14½. Mean thermometer, 33.4°. Rain fall, .63 inches.

MORTALITY BY WARDS.

| Wards. | No. Deaths. | Pop. in 1874. | Percentage. | |
|---|---|---|---|---|
| 1 | 0 | 5,725 | one death in | |
| 2 | 9 | 4,830 | " " | 537 |
| 3 | 14 | 14,861 | " " | 1,061 |
| 4 | 11 | 15,361 | " " | 1,397 |
| 5 | 18 | 20,078 | " " | 1,114 |
| 6 | 29 | 35,916 | " " | 1,238 |
| 7 | 29 | 31,722 | " " | 1,094 |
| 8 | 27 | 29,143 | " " | 1,075 |
| 9 | 33 | 31,654 | " " | 959 |
| 10 | 17 | 17,385 | " " | 1,023 |
| 11 | 15 | 14,022 | " " | 935 |
| 12 | 16 | 16,792 | " " | 1,050 |
| 13 | 14 | 17,892 | " " | 1,278 |
| 14 | 18 | 16,720 | " " | 929 |
| 15 | 57 | 45,545 | " " | 719 |
| 16 | 25 | 21,922 | " " | 877 |
| 17 | 23 | 20,777 | " " | 940 |
| 18 | 21 | 21,392 | " " | 1,319 |
| 19 | 1 | 4,677 | " " | 4,677 |
| 20 | 10 | 8,995 | " " | 900 |
| | 387 | Ratio of deaths to population in 1874, one death in 870⅛. | | |

| | | | |
|---|---|---|---|
| No. deaths in Wards | 387 | Mercy Hospital | 5 |
| Accidents | 18 | Police Station | 1 |
| Bridewell | 1 | St. Joseph's Hospital | 3 |
| Alexian Bros. Hospital | 6 | St. Luke's Hospital | 3 |
| County Hospital | 4 | On cars | 1 |
| Foundlings' Home | 15 | Suicides | 4 |
| Hahnemann Hospital | 1 | | |
| Home for Friendless | 1 | Total | 451 |
| Hospital for Women and Child'n | 1 | | |

### MORTALITY REPORT, CITY OF CHICAGO, FOR THE YEAR 1874.

| Wards, 1874. | January. | February. | March. | April. | May. | June. | July. | August. | September. | October. | November. | December. | Total. | Population, 1874. | One Death in | Deaths in 1,000 Population. |
|---|---|---|---|---|---|---|---|---|---|---|---|---|---|---|---|---|
| 1 | 1 | 4 | 4 | 4 | 1 | .. | 1 | 4 | 4 | 6 | 5 | .. | 34 | 5,725 | 168 1-3 | 5.94 |
| 2 | 4 | 3 | 4 | 3 | 2 | 3 | 6 | 8 | 4 | 1 | 1 | 9 | 48 | 4,830 | 100 3-5 | 9.93 |
| 3 | 15 | 18 | 26 | 33 | 15 | 16 | 27 | 34 | 27 | 13 | 15 | 14 | 253 | 14,861 | 58 2-5 | 17.02 |
| 4 | 12 | 10 | 10 | 10 | 7 | 8 | 16 | 20 | 23 | 10 | 14 | 11 | 151 | 15,361 | 101 4-5 | 9.83 |
| 5 | 19 | 11 | 21 | 17 | 19 | 13 | 32 | 40 | 16 | 26 | 18 | 18 | 250 | 20,078 | 80 1-3 | 12.45 |
| 6 | 51 | 57 | 39 | 53 | 38 | 41 | 132 | 126 | 91 | 47 | 47 | 29 | 751 | 35,916 | 47 5-6 | 20.91 |
| 7 | 41 | 40 | 39 | 43 | 32 | 61 | 185 | 149 | 70 | 59 | 35 | 29 | 783 | 31,722 | 40 1-2 | 24.68 |
| 8 | 30 | 30 | 35 | 21 | 32 | 47 | 138 | 119 | 72 | 48 | 28 | 27 | 627 | 29,143 | 46 1-2 | 21.51 |
| 9 | 38 | 26 | 40 | 24 | 36 | 42 | 110 | 75 | 45 | 29 | 34 | 33 | 532 | 31,654 | 59 1-2 | 16.80 |
| 10 | 16 | 14 | 21 | 13 | 13 | 9 | 27 | 26 | 11 | 12 | 11 | 17 | 190 | 17,385 | 91 3-5 | 10.93 |
| 11 | 18 | 18 | 19 | 11 | 11 | 11 | 29 | 32 | 18 | 11 | 9 | 15 | 202 | 14,022 | 69 4-7 | 14.40 |
| 12 | 16 | 11 | 11 | 15 | 17 | 12 | 45 | 40 | 21 | 24 | 11 | 16 | 239 | 16,792 | 70 1-4 | 14.10 |
| 13 | 19 | 20 | 18 | 13 | 9 | 8 | 39 | 28 | 28 | 13 | 14 | 14 | 223 | 17,892 | 80 1-4 | 12.52 |
| 14 | 16 | 11 | 14 | 13 | 19 | 11 | 41 | 41 | 18 | 24 | 12 | 18 | 238 | 16,720 | 70 1-4 | 14.20 |
| 15 | 87 | 52 | 62 | 35 | 64 | 75 | 234 | 169 | 129 | 65 | 59 | 57 | 1,088 | 45,545 | 41 9-10 | 23.91 |
| 16 | 34 | 32 | 24 | 34 | 43 | 37 | 94 | 68 | 59 | 40 | 23 | 25 | 513 | 21,922 | 42 3-4 | 23.31 |
| 17 | 35 | 21 | 28 | 22 | 28 | 26 | 68 | 72 | 42 | 36 | 17 | 23 | 418 | 20,778 | 49 2-3 | 20.12 |
| 18 | 29 | 23 | 24 | 30 | 33 | 16 | 82 | 65 | 41 | 20 | 21 | 21 | 405 | 21,392 | 52 3-4 | 18.93 |
| 19 | 2 | 6 | 3 | 3 | 4 | 1 | 15 | 7 | 7 | 4 | 4 | 1 | 57 | 4,677 | 82 | 12.20 |
| 20 | 11 | 7 | 11 | 7 | 11 | 3 | 22 | 15 | 6 | 10 | 7 | 10 | 120 | 8,995 | 75 | 13.33 |
| ...... | 494 | 414 | 453 | 404 | 434 | 440 | 1,343 | 1,138 | 732 | 498 | 385 | 387 | 7,122 | 395,409 | .......... | ....... |
| Deaths by accidents, exclusive of Ward mortality. | | | | | | | | | | | | | 874 | .......... | .......... | ....... |
| Reports received after the monthly mortality report was made out | | | | | | | | | | | | | 29 | .......... | .......... | ....... |
| Total. | | | | | | | | | | | | | 8,025 | .......... | .......... | ....... |

Average death percentage by Wards, one death in 55 2-5. Death rate exclusive of Wards, by accidents, etc., one death in 452 2-5. Ratio of deaths, all told, one in 50 1-13, or 20.29 in 1,000.

